# Volumetric‐modulated arc therapy planning using multicriteria optimization for localized prostate cancer

**DOI:** 10.1120/jacmp.v16i3.5410

**Published:** 2015-05-08

**Authors:** Sarah Ghandour, Oscar Matzinger, Marc Pachoud

**Affiliations:** ^1^ Cancer Center – Radiotherapy Department Riviera‐Chablais Hospital Vevey Switzerland

**Keywords:** VMAT, MCO, prostate, treatment planning, deliverability

## Abstract

The purpose of this work is to evaluate the volumetric‐modulated arc therapy (VMAT) multicriteria optimization (MCO) algorithm clinically available in the RayStation treatment planning system (TPS) and its ability to reduce treatment planning time while providing high dosimetric plan quality. Nine patients with localized prostate cancer who were previously treated with 78 Gy in 39 fractions using VMAT plans and rayArc system based on the direct machine parameter optimization (DMPO) algorithm were selected and replanned using the VMAT‐MCO system. First, the dosimetric quality of the plans was evaluated using multiple conformity metrics that account for target coverage and sparing of healthy tissue, used in our departmental clinical protocols. The conformity and homogeneity index, number of monitor units, and treatment planning time for both modalities were assessed. Next, the effects of the technical plan parameters, such as constraint leaf motion CLM (cm/°) and maximum arc delivery time T (s), on the accuracy of delivered dose were evaluated using quality assurance passing rates (QAs) measured using the Delta^4^ phantom from ScandiDos. For the dosimetric plan's quality analysis, the results show that the VMAT‐MCO system provides plans comparable to the rayArc system with no statistical difference for V95% (p<0.01), D1% (p<0.01), CI (p<0.01), and HI (p<0.01) of the PTV, bladder (p<0.01), and rectum (p<0.01) constraints, except for the femoral heads and healthy tissues, for which a dose reduction was observed using MCO compared with rayArc (p<0.01). The technical parameter study showed that a combination of CLM equal to 0.5 cm/degree and a maximum delivery time of 72 s allowed the accurate delivery of the VMAT‐MCO plan on the Elekta Versa HD linear accelerator. Planning evaluation and dosimetric measurements showed that VMAT‐MCO can be used clinically with the advantage of enhanced planning process efficiency by reducing the treatment planning time without impairing dosimetric quality.

PACS numbers: 87.55.D, 87.55.de, 87.55.Qr

## I. INTRODUCTION

Over the past few years, volumetric intensity‐modulated arc therapy (VMAT) has rapidly expanded as an advanced form of external beam radiotherapy.^(1)^ This is due mainly to the equivalent dose distribution quality achieved with VMAT compared with the fixed intensity‐modulated radiation therapy (IMRT) technique, which has the advantage of reducing treatment delivery time.^2^, ^3^ Many commercial treatment planning systems (TPS), such as Eclipse (Varian Medical Systems, Inc., Palo Alto, CA), Monaco (Elekta, Inc., Stockholm, Sweden), SmartArc (Philips, Inc., Andover, MA), and rayArc (RaySearch Laboratories, Stockholm, Sweden), allow for the creation and optimization of dynamic arc plans that meet the clinical goals. Although these systems provide high‐quality plans, the optimal solution generally depends on the planner's time commitment and experience^4^, ^5^ to translate the clinical goals into an optimization problem accounting for the relation between the planning target volume (PTV) coverage and the sparing of the organs at risk (OAR). The process of formulating a clinical treatment plan is generally an iterative time‐consuming process, involving the planner and the radiation oncologist, as the treatment planners may not have *a priori* knowledge of how to modify plan objectives to reach the optimal solution.

Currently, RaySearch Laboratories introduced a new multicriteria optimization algorithm (MCO) for IMRT^6^, ^7^ and VMAT^(8)^ that allows the avoidance of these difficulties and provides a real‐time assessment of the tradeoff between different clinical goals. The idea underlying the MCO formulation is that each anatomical structure is assigned one or several objective functions, and the overall goal is to determine the best possible tradeoff between these objectives using the Pareto optimality concept. The user can navigate through a “Pareto‐optimal” plans database to produce a desired plan that meets the clinical goals.^(9)^ A review of the Pareto optimality optimization concept can be found in Craft.^(10)^

This facilitates the optimization of the different dosimetric parameters and provides multiple solutions in real time, thus eliminating the time‐consuming problem of manual treatment planning due to human interaction in the optimization process. Additionally, the advantage of this new concept of planning is that it allows changing the standard treatment planning workflow to give the radiation oncologist the opportunity to be more involved in the planning process, as well as allow him to navigate to a plan that corresponds to his clinical criteria and that may differ from the one that fulfills the planner's prescribed set of goals.

Recently, many publications have shown the advantage of using the MCO algorithm to generate optimal IMRT treatment plans in terms of dose distribution and planning efficiency. ^11^, ^12^ Although several papers^13^, ^14^ have been published recently that assess the use of the MCO algorithm for VMAT, to our knowledge no clinical assessment of the fully integrated VMAT‐MCO algorithm available in the RayStation treatment planning system has been performed. All of these studies focused on the quality of the dosimetric plans with incomplete information about the machine delivery aspect of these plans. Therefore, in this paper, we describe our experience generating optimal clinically deliverable plans using this algorithm and the Elekta Versa HD Linear Accelerator with the Agility multileaf collimator (MLC) from Elekta (Elekta, Inc., Stockholm, Sweden) for patients with localized prostate cancer. The purposes of this study are thus twofold: first, to perform a dosimetric quality comparison between the VMAT‐MCO plans and benchmark plans generated using the rayArc algorithm based on direct machine parameter optimization (DMPO)^(15)^ and second, to investigate in detail the technical plan parameters to increase the accuracy of the deliverable MCO‐VMAT dose on the treatment machine.

## II. MATERIALS AND METHODS

### A. Prostate cancer cases

The prostate planning study presented in this paper is consistent with the 22991 EORTC ROG trial for localized prostate cancer.^16^, ^17^, ^18^ The clinical target volume (CTV) includes the prostate and seminal vesicles. The PTV extends the CTV using a 0.5 cm margin in all directions, except posteriorly in the rectal–prostate interface region, where a 0.3 cm margin was used. The organs at risk (OARs) are the rectum, bladder, and femoral heads. A 78 Gy dose in 39 fractions was prescribed to cover the PTV. We required that 98% of the PTV received at least 95% of the prescribed dose (Dp); the maximum PTV dose must not exceed 107% of the Dp and must be kept away from the urethra. Dose‐volume histogram (DVH) constraints for OARs were adapted to our clinical constraints: for both rectum and bladder, D20% should not receive more than 70 Gy and D10% should not receive more than 75 Gy. No more than D1% of the bladder volume should receive more than 79 Gy. The maximum dose to the femoral heads did not exceed 48 Gy.

Nine planning CT scans and the target volumes of previously and consecutively treated patients with biopsy‐proven localized prostate cancer who were previously treated with the VMAT‐rayArc were selected from our database and were replanned.

### B. Optimization algorithm: rayArc vs. MCO

The rayArc optimization algorithm is based on the DMPO algorithm.^(15)^ This algorithm employs a multistep process and directly uses controllable machine parameters to optimize the treatment plan arcs. Briefly, the user begins by setting the basic parameters, iteration number, arc length, and couch and collimator angles for an arc. Next, coarse arc segmentation is performed using 24° gantry spacing. VMAT optimization is initialized using fluence maps of these segments. The optimized fluence maps are converted to MLC segments or control points. These segments are distributed to adjacent gantry angles and sorted to approximately minimize the overall leaf travel for the arc. The interpolated segments are then added to achieve the final arc spacing selected by the user. The resulting segments are optimized using all MLC and machine constraints (leaf travel, dose rate, and gantry speed constraints) to satisfy the dose volume objectives. During this process, the optimization variables that are considered are the leaf positions at each control point, the number of monitor units per degree, and the delivery time for each arc. Thus, the resulting optimized plan directly corresponds to the machine delivery settings.

MCO is based on an approximation of the Pareto surface‐based technique^(19)^ which facilitates efficient exploration of tradeoffs between tumor control and sparing normal tissue and is represented by a predetermined number of plans. Briefly, the idea underlying interactive MCO functionality is that a set of Pareto‐optimal plans, which are plans where no criterion can be improved without deteriorating another, are precalculated and stored in a database. Craft and Bortfeld^(7)^ showed that use of 2n plans yields a useful Pareto surface approximation, where n corresponds to the number of the objective cost functions. The user can then navigate in real time through this database using navigation sliders and observe improvement or deterioration for each objective function. Slider movement can cause movement in other sliders, depending on how the objectives are correlated, and an update of the dose distribution and DVH are displayed in real time. The VMAT‐MCO algorithm uses three different algorithms: an algorithm for approximating a convex Pareto surface^(20)^ for fluence map optimization; an algorithm for navigating^(10)^ the discrete Pareto surface representation, which allows the user to select the “best” plan; and a DMPO VMAT optimization algorithm to generate and optimize MLC segments.^(21)^ Notably, navigation through the database corresponds to continuous interpolation^(22)^ between the precalculated plans, and the optimality of the selected interpolated solution will depend on the accuracy of the Pareto‐optimal set. Navigation through the Pareto‐optimal plan database is performed with respect to fluence‐based treatment plans. The navigated fluence‐based plans are obtained using the fluence map optimization (FMO) technique,^(8)^ which reduces the intensity modulation to an attainable level using deliverable VMAT plans. Next, the navigated plan is converted into a deliverable VMAT plan using DMPO to minimize the error between the navigated plan DVH and its best deliverable DVH approximation.^(11)^ More details on the VMAT‐MCO algorithm are provided by Bokrantz.^(8)^

For both algorithms, the VMAT optimization process uses a dose algorithm based on singular value pencil beam kernel decompositions.^(23)^ Intermediate and final dose calculations are performed using a collapsed cone algorithm (CC).^(8)^ The advantage of using an intermediate dose step is that it provides an accurate dose that is used as a background dose during subsequent optimization, which minimizes the discrepancies in computed doses between the pencil beam and CC algorithms.

### C. Optimization functions

In this study on localized prostate cancer cases, the VMAT‐MCO objective optimization was mainly conducted with respect to convex dose‐based functions^(24)^ such as the following: uniform dose, dose falloff, and equivalent uniform dose EUD functions.^25^, ^26^ The DVH‐based functions were used as hard constraints in order to respect the physician constraints discussed in the Materials & Methods section A. Note that the DVH‐based functions are nonconvex in doses due to the volume effect leading to multiple local minima,^(27)^ but studies^(28)^ have shown that they are compatible with gradient‐based optimization algorithms.

For targets, we used the maximum/minimum dose and uniform dose (implemented as a quadratic dose function in RayStation) to create interval limits wherein underdosage and overdosage may be penalized using the same penalty. Further, the dose falloff cost function was used to conform the dose distribution to the target volume by selecting a desired dose level at a given distance from the target. For OARs, such as the bladder and rectum, which exhibit a large volume effect, the EUD function was used to reduce the average dose in these structures that overlap with the PTV.^(6)^ Table 1 shows the standard objectives and constraints that were used for VMAT‐MCO optimization. As the optimal balanced dose distribution for the PTV and OARs depends highly on the patients' geometry,^(29)^ the values of the constraint functions were sometimes adjusted to fulfill the PTV coverage and the sparing of OARs.

**Table 1:** Objectives and constraints used for MCO optimization.

*Objectives*	*Constraints*
PTV: minimum dose 78 Gy	PTV: minimum dose 76 Gy
PTV: uniform dose 78 Gy	PTV: maximum dose 80 Gy
Bladder: Max.EUD=0 Gy (a=1)	Bladder: V75 Gy<10%
Rectum: Max.EUD=0 Gy (a=1)	Rectum: V75 Gy<10%
External‐PTV: dose falloff 74.1 Gy to 0 Low distance 1 cm	
	Femoral Heads (Left & Right): <48 Gy

a PTV=planning target volume; V75=the volume receiving at least 75 Gy; EUD=equivalent uniform dose.

### D. Plan quality evaluation

The quality of the plans was evaluated using the PTV and OAR DVH values already discussed in section A above. Mean doses to the rectum, bladder, and femoral heads were reported, as well as the number of monitor units (MUs) for both algorithms. The conformity index (CI) and homogeneity index (HI) for all plans were assessed using the RTOG definitions^(30)^ as follows:

(1) $$CI=\frac{V_{PI}}{TV},HI=\frac{D_{5}}{D_{95}}$$

where $V_{P1}$ is the prescribed isodose volume, and *TV* is the planning target volume covered by the prescribed isodose. For an ideal plan, *CI* equals 1. $D_{5}$ is the minimum dose that covers 5% of the PTV, and $D_{95}$ is the minimum dose that covers 95% of the PTV.

### E. VMAT settings and plan parameters

We created a benchmark plan for every patient using the rayArc algorithm by manually setting the objective functions' weight. The optimization process was performed iteratively until the clinical goals were reached (i.e., the manual parameters were manually tuned, the optimization and dose calculation processes were performed, the DVH and dose distributions were evaluated, and the process was repeated until the goals were met). For the VMAT‐MCO algorithm, the Pareto surface was approximated by calculating 2n plans, where n corresponds to the number of objective cost functions used during the optimization process; in our case n was equal to 5 (cf. Table 1).

Prostate VMAT plans were generated using the nominal energy of 6 MV and the dual arc feature of 360°. The advantage of using this feature is that the first arc will focus on the left side of the target and the second will focus on the right side, which decreases travel back and forth over the OARs and, thus, the dose to these organs. Tang et al.^(31)^ showed that using multiple arcs yields better quality plans over single arc VMAT with better OAR sparing.

As mentioned in the Materials & Methods section B, the navigation through the Pareto‐optimal plan database is performed with respect to fluence‐based treatment plans without knowledge of the final deliverable dose distribution. Thus, in order to reap the full benefits of the VMAT‐MCO algorithm, the crucial step of finding the optimal technical parameters (e.g., number of arcs, gantry spacing and speed, constraint leaf motion) should be investigated in a way that allows the navigated plan to be deliverable on the machine. This study was necessary to determine the ability of the MCO‐VMAT algorithm to calculate a dose that accurately represents the delivered dose.

The optimization algorithm of RayStation uses three principle parameters: 1) the “constraint leaf motion (cm/deg)” (CLM), 2) the maximum arc delivery time (s), and 3) the gantry spacing (degree). The CLM controls the control points (CPs) aperture shape with a beam angle and should be set depending on the plan's modulation complexity.^(32)^ It is directly related to leaf travel between consecutive CPs, which significantly impacts TPS calculation and dose delivery accuracy.^(33)^

The impact of CP sampling and gantry spacing on optimization was studied by Masi et al.^(32)^ In our study, the plans were optimized and calculated using the gantry angle sampling frequency 3° to compromise between the number of optimizable MLC leaf positions and calculated time.^32^, ^34^ The maximum arc delivery time controls the gantry rotation speed and impacts the MLC speed combined with the CLM. Bedford et al.^(35)^ showed that, when the gantry moves at full speed, the Agility MLC of Elekta can move 0.55 cm/degree, and when the gantry slows down to approximately half the maximum speed, the MLC moves 0.8 cm/degree. Thus, the combination of the two factors CLM and maximum delivery time generally affects the dose delivery accuracy using a linear accelerator. In RayStation, the user can specify these parameter's values independently and *a priori*. Thus, in order to find the optimal deliverable VMAT plans, a set of plans was generated by varying the gantry speeds (3°, 4°, 5°, and 5.5°/s) and the maximum MLC leaf positions between adjacent control points (0.3, 0.5, 0.6, 0.8, and 1 cm/degree). The dosimetric impact of these parameters was assessed using the Delta^4^ phantom (ScandiDos, Uppsala, Sweden) QA passing rates based on the gamma index γ analysis, which was proposed by Low et al.,^(36)^ to compare the calculated and measured doses in terms of the dose difference and distance to agreement (3%,3 mm). The QA passing rate is expressed as the percentage of points with γ<1.

To quantify the correlation between these parameters and the QA passing rate, we used the modulation complexity score (MCS) originally proposed by McNiven et al.^(33)^ for IMRT plans, which was then adapted for VMAT by Masi et al.^(32)^ The MCS computation is based on a combination of the mean values between the adjacent CP of the aperture area variability (AAV) and leaf sequence variability (LSV) weighted by the relative monitor units (MU) delivered between two consecutive CPs and then summed over all CPs in the arc. The MCS is defined as follows:

(2) $$MCS=\sum_{i=1}^{1-1}[(\frac{AAV_{cpi}+AAV_{cpi+1}}{2})\times (\frac{LSV_{cpi}+LSVcp_{i+1}}{2})\times \frac{MU_{CPi,i+1}}{MU_{arc}}]$$

When the plan modulation increases (high CLM), the MCS score decreases, and thus an MCS equal to 1 indicates no leaf motion and no plan modulation complexity. More details on calculating the MCS can be found in Masi et al.^(32)^

## III. RESULTS

**Figure 1:**
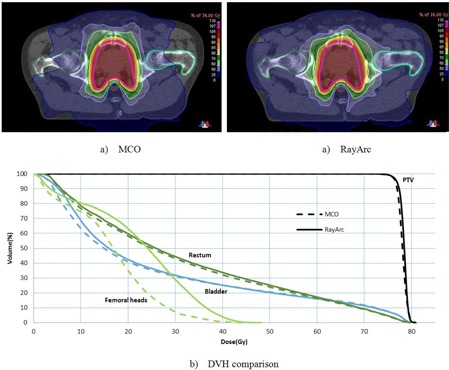
Dose distributions (a) using the MCO and rayArc systems for a typical patient; (b) a comparison of the average DVHs of the MCO and rayArc plans.

All VMAT‐MCO plans achieved the required PTV coverage; on average, 99.6% of the PTV received more than 74.1 Gy (95% of the prescription dose), and the average D1% was 80.2 Gy (<81 Gy). The rectum was spared, as defined by V70Gy, less than 20% (average 9.2%), and it was spared, as defined by V75Gy, less than 10% (average 5%). Figure 1 shows the dose distribution comparison between the MCO and rayArc plans for a typical patient and illustrates the average DVH for PTV, rectum, bladder, and femoral heads for both algorithms. The results show that, for the rectum and bladder, no large differences in the high‐dose regions were detected and slightly better sparing was observed in the low‐dose regions for VMAT‐MCO. However, a significant difference for femoral heads was observed, with a decrease in dose up to 26% with the MCO algorithm. VMAT‐MCO plans use an average of 643 MUs (range 538 to 781) compared with 597 MUs (range 468 to 767) for rayArc plans; therefore, an increase of 8% was observed for MCO compared with rayArc. The mean HIs were 1.04 and 1.03 (σ=0.01), and the mean CIs were 1.26 and 1.25 (σ=0.1) for the MCO and rayArc, respectively. Table 2 shows the average metrics of PTV, OARs, MU, HI, and CI of the MCO and rayArc plans among the nine patients. The results show that both the MCO and rayArc modalities provide a similar or comparable plan quality with no significant differences for PTV coverage and rectum and bladder sparing, although a significant improvement of femoral head sparing was observed with MCO.

**Table 2:** Average results for the nine patients for the different plan metrics.

*Index*	*MCO*	*rayArc*	*Statistical Differences Between MCO and rayArc* ^a^
PTV			
V74.1 Gy (%)	99.59±0.43	99.79±0.12	No difference p<0.01
D1% (Gy)	80.2±0.54	79.94±0.45	No difference p<0.01
CI	1.26±0.09	1.25±0.11	No difference p<0.01
HI	1.04±0.01	1.03±0.01	No difference p<0.01
MU	643 (538‐781)	597 (468‐757)	Different p<0.05
Rectum			
V70 Gy (%)	9.16±3.87	9.68±4.95	No difference p<0.01
V75 Gy (%)	5.00±3.32	5.23±4.07	No difference p<0.01
Dmean (Gy)	31.31±6.45	32.1±8.04	No difference p<0.01
Bladder			
V70 Gy (%)	12.55±9.4	12.22±9.24	No difference p<0.01
V75 Gy (%)	9.49±8.6	9.36±8.19	No difference p<0.01
Dmean (Gy)	24.01±9.2	25.38±10.26	No difference p<0.05
Femoral Heads			
D1% (Gy)	35.88±5.37	42.69±1.73	Different p<0.01
Dmean (Gy)	17.1±3.62	23.11±3.42	Different p<0.01
Healthy Tissue			
Dmean (Gy)	5.53±1.42	5.94±1.63	Different p<0.01

^a^ Statistical difference between the MCO and rayArc systems with p<0.01 or p<0.05.

PTV=planning target volume; V[n]Gy=volume receiving at least nGy; Dmean=the mean dose in Gy that the volume received; CI=Conformal Index; HI=Homogeneity Index;MU=monitor units.

**Figure 2:**
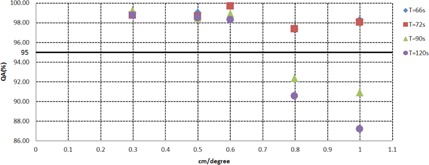
QA passing rates as a function of the arc delivery time and CLM.

The impact of planning parameters on the VMAT dosimetric accuracy was explored by plotting the average QA passing rates for the plans versus the CLM and the arc delivery time, as discussed in the Material & Methods section E. All plans were measured on the same day to avoid uncertainty due to variations of phantom setup. Figure 2 shows that the average QA passing rates of all VMAT plans calculated using the maximum delivery times of 66, 72, 90, and 120 s and CLM values between 0.3 and 0.6 cm/degree were greater than 95%. However, we observed that the other plans generated with CLMs greater than 0.8 cm/degree and T=66/72 s passed with an average of 97.5%, and a significant decrease in QA passing rate was observed for the other plans generated with CLMs greater than both parameters — 0.8 cm/degree and T of 90 s, with an average of 90.2% (σ=2.19%). These results show that higher CLMs (>0.8 cm/degree) enhance the importance of the arc delivery time. Consequently, it was necessary to develop an in‐house, quality‐assurance method that compares the leaf positions per degree calculated in the TPS with data directly extracted from the linear accelerator to evaluate the accuracy of the delivered dose compared to the calculated dose. 3, 4 illustrate the cumulative frequency distribution of leaf movement as a function of the CLM for plans generated with an arc delivery time equal to 66 s and 120 s, respectively, for different CLMs (0.3, 0.5, 0.8, and 1 cm/degree). The results show good agreement between the TPS and linear accelerator for plans generated using CLMs equal to 0.3 and 0.5 cm/degree for the different arc delivery times (66 s and 120 s). However, plans generated with a CLM greater than 0.8 cm/degree showed poor agreement between both plots (the TPS and linear accelerator), mainly for the maximum delivery time of 120 s, where the machine does not respect the CLM constraint fixed in the TPS. The difference could be explained by the fact that plans generated with 66 s are constrained by a CLM equal to 0.65 cm/degree due to the maximum MLC speed (3.5 cm/degree) modeled in the TPS that is kept unconstrained for plans generated with 120 s and CLMs greater than 0.8 cm/degree, thus allowing for leaf movement over a considerable distance.

**Figure 3:**
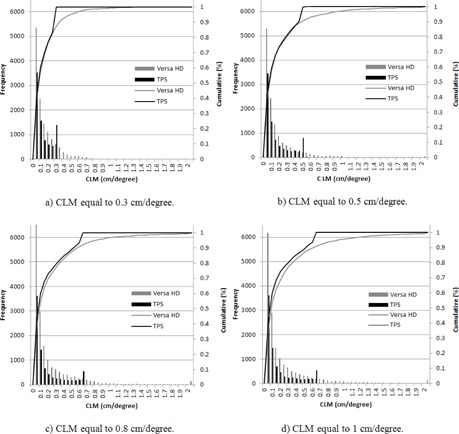
Cumulative frequency distributions for leaf movement as a function of the CLM for the arc delivery time of 66 s.

**Figure 4:**
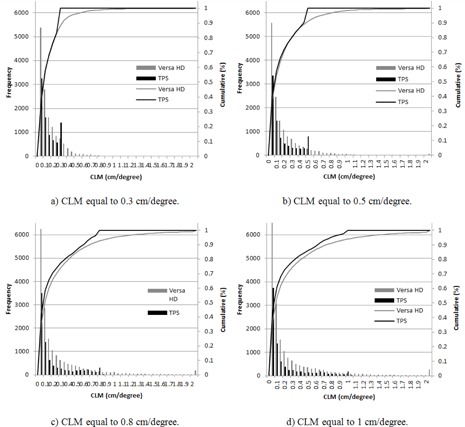
Cumulative frequency distributions of leaf movement as a function of the CLM for the arc delivery time of 120 s.

**Figure 5:**
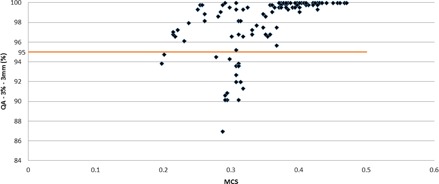
QA passing rates as a function of the MCS score value for each calculated plan. The horizontal line indicates a QA passing rate of 95%.

A quantitative MCS score was generated for the plans to evaluate their complexity and correlation with the QA passing rate. Figure 5 illustrates the relationship between the plan complexity and the QA passing rate, which was previously expressed as a function of the plan's parameters. The results show that all plans with an MCS value greater than 0.35 yielded a passing rate greater than 95%, and when the modulation increased (MCS value below 0.3), the agreement uncertainty between the calculated and measured doses significantly increased. In our study on localized prostate cancer undergoing standard fractionation, a delivery time of 2.4 minutes (72 s for each arc) with a CLM =0.5 cm/degree appears to be a good compromise between plan quality and QA passing rate.

## IV. DISCUSSION

In this paper, we evaluated the VMAT‐MCO algorithm for clinical use by assessing the quality of the final dosimetric plans compared with the rayArc algorithm. MCO is an efficient tool for exploring tradeoffs between conflicting objective functions through a real‐time updated graphic view, and it optimizes clinical workflow by involving physicians in the treatment planning process. We performed a treatment planning study on nine patients with localized prostate cancer to demonstrate the capability of the VMAT‐MCO optimization algorithm to generate plans with the same PTV coverage as the rayArc, but with slightly improved sparing of the OARS including the bladder and rectum, and a significant decrease in the dose for the femoral heads (up to 26%). This is due to the use of the Dose Fall‐off function (including the femoral heads) which has the same weight or importance as the other functions (Max EUD) of bladder and rectum in MCO optimization process. Notably, both algorithms decreased the dose to the rectum and bladder to below our treatment planning constraints. The homogeneity and conformity indexes were approximately the same for both systems. However, the MCO system tended to yield more MUs (8%) compared with the rayArc, which we consider with no clinical effect.

Many papers^4^, ^5^ have shown that plan quality based on minimizing a weighted sum of the objectives (i.e., rayArc) depends on the planner's experience and, especially, time spent optimizing a plan. In our case, for the treatment planner, prostate cases took an average of 75.39 min (σ=14.35 min) with rayArc due to the iterative optimization process, compared with 19.86 min (σ=1 min) for MCO. This difference is due to the database generation of the MCO system, which is completely independent of the user and took 5.72 min on average (σ=0.62 min) plus the time necessary for dose calculation, which was 14.13 min (σ=1.16 min). However, the difficulty with MCO is defining the objectives/constraints and selecting their formulation, which directly affects the Pareto surface computation. To generate optimal clinical plans using the MCO system, we combined convex and nonconvex cost functions that are suitable for the optimization algorithm.

In order to fully benefit from the MCO technique and to generate a deliverable plan that is similar to the navigated plan, we performed a detailed study to find the optimal combination of the technical planning parameters, such as the constraint leaf motion (CLM) and maximum delivery time. Thus, the impact of these parameters on the deliverable VMAT plan was assessed by plotting the QA passing rates (%) as a function of these two parameters. Further, the modulation complexity score (MCS) developed by Masi et al.^(32)^ was used to link the CLM, arc delivery time, and QA passing rate of the Delta^4^ from ScandiDos. The results showed that a delivery time of 2.4 minutes (72 s for each arc) with a CLM equal to 0.5 cm/degree is a good compromise for prostate cancer patients undergoing standard fractionation. Therefore, we concluded that further increasing the combination of the delivery time and CLM beyond 90 s and 0.8 cm/degree, respectively, is not beneficial. This combination yields an MCS threshold equal to 0.35, where the percentage of plans with a QA passing rate greater than 95% (3%/3 mm) is 100%. Thus, the MCS allows to link delivery and complexity of plans, providing useful information for the optimization of patients' QA workflow.

## V. CONCLUSIONS

We have studied the dosimetric plan difference between the VMAT‐MCO system and the standard iterative optimization process for the rayArc in nine localized prostate cancer patients undergoing standard fractionation treatment. The VMAT‐MCO algorithm appears to be a valuable available tool for the RayStation treatment planning system and provides high‐quality plans comparable to the rayArc in terms of PTV coverage and sparing of the OARs.

## ACKNOWLEDGMENTS

The authors thank Neil Dodd, Elekta GmbH and RaySearch Laboratories for their continuous technical support during the project.

## Supporting information

Supplementary Material
