# Supplementary material for: Volumetric‐modulated arc therapy planning using multicriteria optimization for localized prostate cancer
Source: J Appl Clin Med Phys. 2015 May 8;16(3):258–69. doi: 10.1120/jacmp.v16i3.5410 (PMC5690115; doi:10.1120/jacmp.v16i3.5410)
Supplement: Supplementary file 1 — Supplementary Material [file ACM2-16-258-s001.docx]

Volumetric modulated arc therapy planning using multicriteria optimization for localized prostate cancer

**Sarah Ghandour, PhD, Oscar Matzinger, MD, and Marc Pachoud, PhD**

*Cancer Center – Radiotherapy Department, Riviera-Chablais Hospital, Vevey Switzerland*

*Corresponding author: Marc Pachoud, Ph.D., Avenue de la Prairie 1, CH-1800 Vevey, Switzerland*

*marc.pachoud@hopitalrivierachablais.ch*

Running title: VMAT multicriteria optimization
